# Potential Antidiabetic Activity of Extracts and Isolated Compound from *Adenosma bracteosum* (Bonati)

**DOI:** 10.3390/biom10020201

**Published:** 2020-01-29

**Authors:** Ngoc Hong Nguyen, Quang Thang Pham, Thi Ngoc Han Luong, Hoang Khai Le, Van Giau Vo

**Affiliations:** 1 CirTech Institute, HCMC University of Technology (HUTECH), Ho Chi Minh City 700000, Vietnam; 2 Institute of Applied Science, HCMC University of Technology (HUTECH), Ho Chi Minh City 700000, Vietnam; pquangthang1@gmail.com (Q.T.P.); ngochanlt96@gmail.com (T.N.H.L.); lehoangkhai2016@gmail.com (H.K.L.); 3 Bionanotechnology Research Group, Ton Duc Thang University, Ho Chi Minh City 700000, Vietnam; 4 Faculty of Pharmacy, Ton Duc Thang University, Ho Chi Minh City 700000, Vietnam

**Keywords:** α-glucosidase inhibition, anti-diabetic, extract, isolated compound, STZ

## Abstract

*Adenosma bracteosum* Bonati. (*A. bracteosum*) has been used in traditional and modern medicine in Vietnam for curing hepatitis. In this study, ethanol and aqueous extracts of *A. bracteosum* were evaluated for their α-glucosidase inhibitory activities and anti-hyperglycemic effects on glucose loaded hyperglycemic and streptozotocin (STZ) induced diabetic mice. The α-glucosidase inhibition of the extracts was evaluated by colorimetric assays, and the anti-diabetic activity was tested on a STZ-induced diabetic mice model. The ethanol and aqueous extracts showed a significant α-glucosidase inhibitory activity, which was more effective than acarbose at the same concentration. In the STZ-induced diabetic mice, both extracts showed a strong anti-hyperglycemic activity, with the group receiving 50 mg/kg of ethanol extract and the group receiving 50 mg/kg of aqueous extract presenting 64.42% and 57.69% reductions, respectively, in the blood glucose levels when compared with the diabetic control group, on day 21 (*p > 0.05*). Isoscutellarein-8-O-β-D-glucopyranoside (IG) was identified from the ethanol extract, which showed a strong inhibitory activity against α-glucosidase, with a ten times higher potency compared with the positive control acarbose. The anti-hyperglycemic effect of IG was effectively similar to the standard drug, glibenclamide, at the same dose of 10 mg/kg (*p* > 0.05). These results indicated that *A. bracteosum* has a great antidiabetic potential.

## 1. Introduction

Diabetes mellitus, or simply diabetes, is a syndrome characterized by high blood glucose levels that result from defects in the body’s ability to produce and/or use insulin. About 422 million people worldwide have diabetes, particularly in low- and middle-income countries, making diabetes mellitus one of the leading causes of death [1,2]. Chronic hyperglycemia during diabetes causes the glycation of proteins, which in turn leads to complications (damage) affecting the eyes, kidneys, nerves, and arteries [3]. Given the important role of oxidative stress in the pathogenesis of many clinical conditions and aging, antioxidant therapy could positively affect the natural history of several diseases. Hyperglycemia activates a particular metabolic route that involves diacylglycerol, protein kinase C, and NADPH-oxidase, culminating in reactive oxygen species (ROS) [4]. It is suggested that ROS is induced by hyperglycemia in diabetic patients through mitochondrial respiratory chain enzymes, xanthine oxidases, lipoxygenases, cyclooxygenases, nitric oxide synthases, and peroxidases [5,6,7].

Medicinal plants continue to contribute significantly to developing modern prescription drugs by providing lead compounds upon which the synthesis of new drugs can be made. Therefore, the hypoglycemic activity of a number of plant extracts has been evaluated and confirmed in animal models [8,9,10]. Many recent studies have revealed that plant-based formulations can contribute to a better management of diabetes, as certain phytochemicals exert antioxidant, anti-inflammatory, and glucose-lowering effects [10,11,12,13]. In Vietnam, *Adenosma bracteosum* Bonati. (Scrophulariaceae family) is indicated in the traditional treatment of liver diseases [14,15]. Previously, we have demonstrated the antioxidant, anti-gout, hepato-protective, and anti-hyperglycemic activities of *A. bracteosum* extract; the major components of essential oil from the *A. bracteosum* aerial part were thymol, linalool, and (E)-β-farnesene [15]. The present study was further undertaken to investigate the antidiabetic activity using various in vitro and in vivo models designed to stimulate specific antidiabetic targets.

## 2. Materials and Methods

### 2.1. Chemicals and Reagents

Ethanol (Sigma Aldrich) and distilled water were used for the extraction of the plant materials. ABTS (2,2′-azino-bis-(3-ethylbenozothiazonline-6-sulfonic acid)), 2,2-diphenyl-1-picrylhydrazyl (DPPH), p-nitrophenyl-α-D-glucopyranoside (pNPG), α-glucosidase, gallic acid, and rutin were purchased from Sigma-Aldrich (St. Louis, USA). Folin-ciocalteu reagent, acarbose, gallic acid, rutin, silicagel, dimethyl sulfoxide, and methanol were obtained from Merck (Darmstadt, Germany). Glibenclamide and streptozotocin were purchased from Hi-media (Mumbai). Ascorbic acid was from Scharlau (Scharlab). All of the other chemicals were of analytical grade.

### 2.2. Preparation of Plant Extracts and Isolation

The plant of *A. bracteosum* was collected at Ba Den Mountain, Tay Ninh province, Vietnam, in December 2017. The sample was identified by Assoc. Prof. Tran Hop, Ho Chi Minh City University of Natural Science, Vietnam. The aerial parts were shade-dried, pulverized to a medium-size powder, sieved with a 1-mm diameter mesh, and macerated by ethanol 90% for 48 h at room temperature. After filtration, the solvents were removed under reduced pressure at 35 °C to obtain a crude ethanol extract. The aqueous extract was prepared by boiling the dried and coarsely powdered material with water in a flask fitted with a reflux condenser for 30 min. This extract was then filtered and concentrated, as described above. The extracts were used for the in vivo and in vitro experiments.Ethanol extract showed strong biological activities and was subjected to silica gel column vacuum chromatography and elution with hexane in chloroform (100:0–0:100) to give nine fractions. These fractions were used to test for their antioxidant effects. The seventh fraction showed a strong antioxidant activity, and this fraction was further purified by column chromatography eluted with ethyl acetate and methanol (10:1) to give a yellow powder compound. To identify the compound, the molecular weight and the structure was analyzed by mass spectrometry (MS), ^1^H-NMR, ^13^C-NMR, heteronuclear multiple-bond correlation (HMBC), and heteronuclear single quantum coherence (HSQC) spectroscopy.

### 2.3. In Vivo Confirmatory Studies

#### 2.3.1. Experimental Animals

Swiss albino mice weighing 20–25 g (6–8 weeks old, average body weight of 25 g) were provided by Ho Chi Minh Pasteur Institute in Vietnam. The animals were divided into groups, each comprising 6–10 animals and maintained under laboratory conditions (temperature 24–28 °C, relative humidity 60%–70%, and 12-h dark:light cycles), and with pelleted food and water available ad libitum.

#### 2.3.2. Preliminary Phytochemical Investigation

Phytochemical analysis of the samples was used for the identification of constituents, using procedures previously described [9]. The presence of alkaloids was tested by Mayer, Dragendorff, and Bouchardat’s reagent; phenolics and tannin were tested with 2% FeCl_2_ and 1% gelatine in 10% NaCl; the flavonoids were tested with a Shinoda test and alkaline reagent; triterpenoids were tested with the Liebermann–Burchard test; steroids were tested with a Salkowski test; and saponins were tested by a foam test with distilled water.

#### 2.3.3. Determination of Total Phenolic Content

The total phenolic content was assessed using the Folin–Ciocalteu method [16]. First, 1 mL of extract (0.05:1 mg/mL) was mixed with the Folin–Ciocalteu reagent (5 mL, 1:10 v/v) and aqueous Na_2_CO_3_ (4 mL, 7.5%). The mixture was incubated for 30 min at 40 °C, and the absorbance of the samples was determined by colorimetry at 765 nm. The amount of total phenols is expressed based on mg of gallic acid equivalent per gram of extract (mg GAE/g extract).

#### 2.3.4. Determination of Total Flavonoid Content

The total flavonoid contents were estimated using the previously reported method [17]. First, 2 mL of extract was added into 2 mL AlCl_3_ (2%)/ethanol. The mixture was incubated for 1 h at 25 °C. The absorbance was obtained at 420 nm. The total flavonoid contents were expressed as mg of rutin equivalents per gram of extract (mg RE/g extract), using the calibration curve with rutin.

#### 2.3.5. Free Radical Scavenging Activity Assay

The 2,2-Diphenyl-1-picrylhydrazyl (DPPH) assay of the samples was estimated using the reported method [12]. First, 50 μl of sample solutions at different concentrations were mixed with 2 mL of methanol solution of DPPH• radical (6 × 10^−5^ M in 80% methanol). The absorbance reading at 515 nm was determined after 16 min for all of the samples. All of the tested samples were performed in triplicate. The percentage of inhibition of the DPPH• radical was determined according to the following equation [18]:

% Inhibition = [(A_C(0)_ − A_S(t)_)/A_C(0)_)] × 100 (1),

where A_C(0)_ is the absorbance of the control (t = 0 min) and A_S(t)_ is the absorbance of the sample (t = 16 min). The sample concentration obtaining 50% inhibition (IC_50_) was calculated from the graph against the concentration of the samples, and ascorbic acid was used as a reference.

#### 2.3.6. ABTS Radical Scavenging Assay

The radical scavenging activity of the samples was determined according to a previously described procedure with slight modifications [19]. Briefly, ABTS was dissolved in water (7 mM). The radical cation ABTS was generated by reacting the ABTS solution with potassium persulfate (2.45 mM). The mixture was allowed to stand in the dark for 12 h at room temperature before use. Afterward, the ABTS solution was diluted with ethanol and equilibrated at 30 °C. After the addition of 1.0 mL of diluted ABTS solution to 10 mL samples, the absorbance reading at 734 nm was taken at 30 °C after 6 min. IC_50_ was calculated from the graph; ascorbic acid was used as the reference.

#### 2.3.7. In Vitro α-glucosidase Inhibition Assay

The α-glucosidase inhibitory activity was measured as described by previously [20], with slight modifications. Briefly, the mixture of 60 μL of the sample solution and 50 μL of phosphate buffer (0.1 M) at pH 6.8 containing the α-glucosidase solution (0.2 U/mL) was incubated in 96-well microplates for 20 min at 37 °C. After pre-incubation, 50 μL of 5 mM p-nitrophenyl-α-D-glucopyranoside (pNPG) solution in phosphate buffer (0.1 M) at pH 6.8 as the substrate was placed to the mixture (each well) and incubated at 37 °C for 20 min. The reaction was stopped at each well by adding 160 μl of 0.2 M NaCO_3_. The absorbance (A) was measured by the micro-plate reader at 405 nm, and absorbance was compared to the control, which had 60 μl of a buffer solution in place of the extract.

The α-glucosidase inhibitory activity was calculated as follows:

inhibition (%) = (A_control_ − A_sample_) × 100/A_control_ (2)

The concentration of the sample and the reference required to inhibit 50% of the α-glucosidase activity and was determined by the graph plotting against the percentage of inhibition.

#### 2.3.8. Toxicity Determination of Extracts

The oral acute toxicity study of ethanolic and aqueous extracts of *A. bracteosum* were evaluated according to previous studies [21,22], where the limit test dose of 3000 mg/kg was used. Six groups of ten mice were treated with doses (1000, 2000, and 3000 mg/kg, oral/enteral) of each of the ethanol and aqueous extracts, while a control group received distilled water containing 1% dimethyl sulfoxide (DMSO). The animals were observed for any toxic effect for first 1 h after the treatment period. The animals were further investigated for a period of 6 h and 24 h for any toxic effects.

#### 2.3.9. Evaluation of Extracts in Streptozotocin-Induced Diabetic Mice

Experimental diabetes in mice was induced by a single intra-peritoneal injection of 150 mg/kg body weight of streptozotocin (STZ), which was prepared using fresh cold citrate buffer pH 4.5. The control animals, receiving only citrate buffer, had a pH 4.5. The blood glucose level was checked after 72 h of STZ injection. Mice with serum glucose levels of 200 mg/dL were considered diabetic and were used for the study [23,24]. The diabetic mice were divided into four groups of six animals in each group (Groups II–V). Group I: the normal control was given water. Group II: the diabetic control was given distilled water. Group III: the diabetic animals were given glibenclamide (10 mg/kg). Group IV: the diabetic animals were given ethanol extract at a dose of 50 mg/kg. Group V: the diabetic animals were orally given aqueous extract at a dose of 50 mg/kg. The freshly prepared solutions of drug and extracts were orally administered daily for 21 days. The blood glucose levels were tested weekly on the overnight fasted animals. The blood glucose levels of all of the animals (from the tail vein) were measured on days 1, 7, 14, and 21, respectively.

#### 2.3.10. Anti-Hyperglycemic Assay on Glucose Loaded Mice

Glucose tolerance test was executed according to the described method [25], with slight modifications. Briefly, albino mice of either sex were randomly divided into seven groups (six animals each group). Group I: normal control, received distilled water. Group II: mice received standard drug glibenclamide, 10 mg.kg^−1^ weight body. Groups III and IV: mice were treated with aqueous extract of *A. bracteosum* at doses of 40 mg/kg and 50 mg/kg body weight, respectively. Group V and VI: mice were treated with ethanol extract at doses of 40 and 50 mg/kg body weight, respectively. Group VII: mice were treated with an isolated compound at the same dose as glibenclamide (10 mg/kg). All of the treatments were given orally. After one hour, all of the mice were orally treated with 2 g/kgof glucose. The blood samples were collected two hours after the glucose administration. The serum was separated, and the blood glucose levels were estimated.

#### 2.3.11. Statistical Analysis

All of the assessments were performed in triplicate. A one-way analysis of variance (ANOVA) with a Fisher’s Least Significant Difference was performed to determine the significant differences between the parameters, using SAS 9.4 software. The means and standard errors were calculated. Differences among the mean values of the various parameters were determined by the least significant difference test. A probability level of *p* < 0.05 was used in testing the statistical significance of all of the experimental data.

## 3. Results and Discussion

### 3.1. Total Phenolic and Total Flavonoid Content

The extracts were tested for the presence of active substances such as flavonoid, tannin, essential oil, triterpenoids, steroid, and saponins. As shown in Table 1, *A. bracteosum* contains a significant amount of phenolic and flavonoid content. Phenolic compounds play an important role in the biological activity of the plant, and flavonoids are considered to possess strong antioxidant activities among phenolic compounds. The total phenolic and total flavonoid contents vary because of the polarity of the solvents. The phenolic content and flavonoid content were both higher in the ethanol extract (241.47 ± 4.47 mg GAE/g extract and 101.99 ± 4.48, respectively). The phytochemical analysis showed that the ethanol extract of *A. bracteosum* possessed high total phenolic and flavonoid contents, which confirmed its antioxidant activity (Table 2). It was found that the phenolic compounds or phenolic enriched extracts from several plants related to the carbohydrate digestion of enzymes were inhibited significantly [26,27].

### 3.2. Chemical Structure Determination of the Separated Compound

To identify the isolated compound, the molecular weight and the structure were analyzed by MS, ^1^H-NMR, ^13^C-NMR, HMBC, and HSQC spectroscopy.

**Figure 1:**
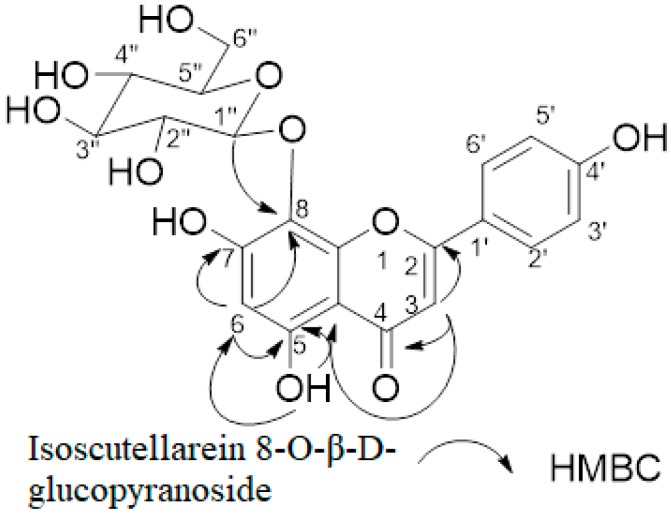
Significant heteronuclear multiple-bond correlations of isoscutellarein-8-O-β-D-glucopyranoside (IG)

ESI-MS m/z: 447.08 [M-H]-^1^H-NMR (500 MHz, DMSO-*d*_6_): δ 12.74 (1H, s, OH), 8.10 (1H, d, *J.* = 8.5 Hz, H-2′/6′), 6.92 (1H, d, *J.* = 9.0 Hz, H-3′/5′), 6.64 (1H, s, H-3), 6.00 (1H, s, H-6), 4.49 (1H, d, *J.* = 8.0 Hz, H-1”), 3.68 (1H, dd, *J.* = 12.0 Hz, 2.0 Hz, H-6”a), 3.61 (1H, dd, *J.* = 12.0 Hz, 4.0 Hz, H-6”b), 3.34 (1H, m, H-5”), 3.24 (1H, m, H-3”, H-4”), 3.15 (1H, m, H-2”). ^13^C-NMR (125 MHz, DMSO-d_6_): δ 181.3 (C-4), 163.3 (C-2), 163.2 (C-7), 161.4 (C-4′), 157.9 (C-5), 149.8 (C-8a), 129.3 (C-2′/C-6′), 122.0 (C-1′), 116.3 (C-5′/C-3′), 108.1 (C-1”), 106.7 (C-4a), 102.3 (C-3), 101.7 (C-6), 77.7 (C-5”), 76.9 (C-3”), 74.6 (C-2”), 69.6 (C-4”), and 61.1 (C-6”). These results were in concordance with the reported results [21]. Figure 1 shows the chemical structure and HMBC correlations of the pure compound isolated from ethanol extract (for the ^1^H, ^13^C, HMBC, and HSQC spectra, please see Figures S1–S5). Therefore, the target compound was identified as isoscutellarein 8-O-β-D-glucopyranoside (IG). The effect of the IG-isolated flavonoid was tested on the α-glucosidase inhibition and anti-hyperglycemic effect on the glucose-loaded mice.

### 3.3. In Vitro α-Glucosidase Inhibition Assay

**Figure 2:**
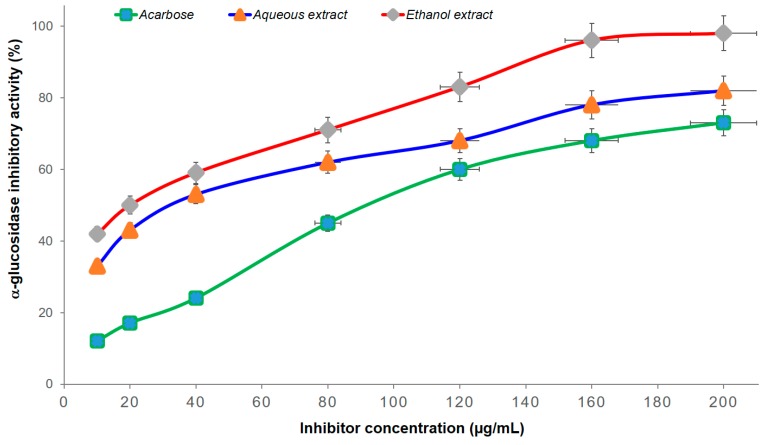
α-Glucosidase inhibition of extracts and acarbose at different concentrations.

The inhibitory effects of the extracts against α-glucosidase were compared with those of acarbose. The results in Figure 2 and Table 3 show that the α-glucosidase inhibitory activity increased with the concentration enhancement (from 10 to 200 µg/mL) of both the extracts and acarbose. At the same concentrations, both extracts were more effective than acarbose. The IC_50_ values of the ethanol extract (26.55 µg/mL) and aqueous extract (42.63 µg/mL) were lower than those of acarbose (87.94 µg/mL). Therefore, the strong inhibition of α-glucosidase and the anti-hyperglycemic effect of both extracts from *A. bracteosum* with α-glucosidase inhibitory activities were obviously higher than those of acarbose (Figure 2). This study provides the first report of the α-glucosidase inhibitory and anti-diabetic effects of ethanol and aqueous extracts from *A. bracteosum*. It has long been a source of exogenous antioxidants, which play important roles in ROS metabolism and the avoidance of uncontrolled oxidation of essential biomolecules, as a result of their wide range of biological activities [8]. Moreover, they are known to interact with other physiological antioxidants, such as ascorbate or tocopherol, and to synergistically amplify their biological effects [28].

### 3.4. Effect of Plant Extracts on Blood Glucose Levels in STZ-Induced Diabetes in Mice

**Figure 3:**
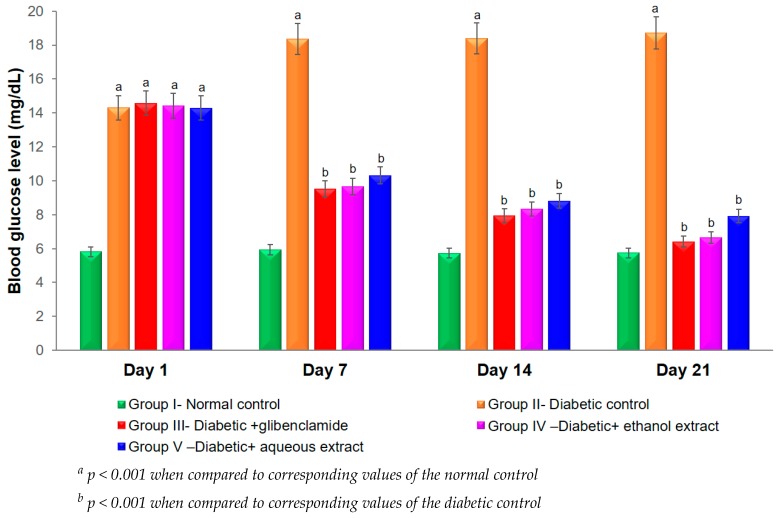
Effects of both extracts from *A. bracteosum* on blood glucose level (mg/dL) in streptozotocin (STZ) induced diabetic mice.

STZ is commonly chosen in experimental animals for the reproducible induction of type I diabetes mellitus. The cytotoxic action of the STZ-diabetogenic agent is mediated by reactive oxygen species by attacking membranes and biological materials, which leads to diabetic complications [29]. The anti-hyperglycemic activity of both extracts was examined in STZ-induced diabetic mice. The hypoglycemic effect of plant extracts in STZ-diabetic mice is presented in Figure 3. The administration of STZ (150 mg/kg, i.p.) induced diabetic mice with blood glucose levels were over 18 mmol/L on day 7 after the STZ injection. From days 7 to 21, the plant extract treated group was heterogeneously significant when compared with the STZ treated diabetic control group (*p* < 0.01). Group IV, receiving ethanol extract, and Group V, receiving aqueous extract at a dose of 50 mg/kg, showed a significant action on the blood glucose level in STZ-induced diabetic mice. Group III, receiving 10 mg/kg drug glibenclamide, and group IV showed homogeneously significant activities on days 7, 14, and 21 (*p* > 0.05). The maximum glucose lowering in the treated diabetic mice (Groups III, IV, and V) was exhibited on day 21. Groups III and IV presented 65.76% and 64.42% reductions in blood glucose level (when compared with Group II), respectively (*p* < 0.05). Oxidative stresses play important roles in initiating beta-cell damage and insulin resistance [30]. The mechanisms of the diabetic complications that contribute to increased oxidative stress in diabetes include metabolic stress, auto-oxidative glycosylation, and non-enzymatic glycosylation [8].

In the above assays, the ethanol extract of *A. bracteosum* had higher α-glucosidase inhibitory activities and anti-hyperglycemic effects than those of the aqueous extract. Therefore, the ethanol extract was further studied for an isolating compound. Anti-oxidants have been prescribed to prevent the progressive impairment of pancreatic cell function and consequently reduce the development of type-2 diabetes [31]. The result of the column chromatography was that a yellow compound was isolated from this extract.

### 3.5. Free Radical Scavenging Determined by DPPH and ABTS Assays Of Extracts and IG Compound from *A. bracteosum*

DPPH is a free radical that can accept an electron of hydrogen. According to the results in Table 2, the IC_50_ values of the ethanol, aqueous extracts, and IG were 6.61, 10.12, and 11.93 μg/mL, respectively, which inhibited the DPPH radical by 2.29, 3.50, and 4.13 times less than of ascorbic acid, respectively. In Table 3, the IC_50_ value of ethanol, aqueous extracts, and IG were 1.97, 2.13, and 2.18 times lower than of ascorbic acid (11.14 ± 0.04 μg/mL), respectively, indicating a significant antioxidant activity of both extracts from *A. bracteosum.* The DPPH and ABTS scavenging activities of the crude ethanol extract with IC_50_ values were only 2.29 and 1.96 times lower than that of the pure ascorbic acid. It may be that the components of the polyphenol and flavonoid from this plant play a crucial role in the antioxidant, inhibition of α-glucosidase, and potential anti-diabetic effects, which are presented in this study.

### 3.6. In Vitro α-Glucosidase Inhibition of IG

As shown in Table 3, the results from the in vitro anti-diabetic effect of the IG compound from *A. bracteosum* with an IC_50_ value of the α-glucosidase inhibitory activity (1.40 µg/mL) displayed a more potent inhibition than the standard anti-diabetic drug acarbose (87.94 µg/mL). The standard acarbose had IC_50_ values of 106.02 µM, while the isolated compound had IC_50_ values of 3.11 µM. The IG compound was the most potent inhibitor of α-glucosidase, followed by the ethanol extract and aqueous extracts. In type-2 diabetes, postprandial hyperglycemia causes oxidative stress and endothelial dysfunction, which lead to diabetic complications [32]. Pancreatic α-amylase and intestinal α-glucosidase are two key carbohydrate digestive enzymes, and elevate the levels of postprandial blood glucose. A method for the effective reduction of postprandial hyperglycemia with the inhibition of α-glucosidase or α-amylase activities is important in the prevention of type-2 diabetic complications. The inhibitors of both of these enzymes, such as acarbose, voglibose, and miglitol, have strong inhibitory activities, but may also have undesired effects [17]. Therefore, the study of inhibitors from natural sources is essential for the control of postprandial hyperglycemia and for the prevention of diabetic complications with minimal side effects.

### 3.7. Oral Glucose Tolerance Tests for the Eevaluation of the Anti-Hyperglycemic Activity

Streptozotocin (STZ)-induced diabetic animal models are useful platforms for understanding β cell glucotoxicity in diabetes. Hence, the effects of *A. bracteosum* extracts on the oral glucose tolerance test in normal and glucose-induced insulin secretion in diabetic mice was examined. Animals treated with both ethanol and aqueous extracts, separately, did not show any change in their behaviors. Their body weights and food consumption were no significant different when compared to the control group. Therefore, it was inferred that the ethanol and aqueous extracts of *A. bracteosum* may be safe for experimental animals at the tested concentrations. Table 4 indicates that the ethanol and aqueous extracts and IG compound of *A. bracteosum* lowered the serum glucose levels significantly when compared to the control group. The anti-hyperglycemic activities of the Groups IV and VI (animals receiving both extracts) at a dose of 50 mg/kg, and Group VII (animals receiving IG) at a dose of 10 mg/kg, were informed with a 51.20%, 54.52%, and 47.30% inhibition, respectively (*p* < 0.01), while the standard drug, glibenclamide, produced a 56.68% inhibitory activity at a 10 mg/kg dose. The anti-hyperglycemic effect in animals of the IG compound was found to be comparable to that of glibenclamide. The results showed that the group receiving the glibenclamide drug and the group receiving IG (6.35 ± 0.11 mmol/L), were effectively similar to the standard drug, glibenclamide (5.91 ± 0.38 mmol/L), at the same dose of 10 mg/kg (*p* > 0.05).

The hypoglycemic effect studied in the glucose-loaded hyperglycemic mice displayed that both extracts of *A. bracteosum* lowered the serum glucose levels remarkably when compared to Group I (received glucose, but not treated with the sample or drug) at all of the doses examined in a dose-dependent manner (Table 4). STZ induced hyperglycemia, with a blood glucose increase of over 315% (over 300 mg/dL on seventh after injection) when compared with the normal control. The oral treatment of both extracts in STZ induced diabetic mice showed a significant blood glucose reduction on days 7, 14, and 21 (Figure 3). The hypoglycemic activity of the animals receiving 50 mg/kg ethanol extract was similar to the animals receiving 10 mg/kg glibenclamide drug (known as a glyburide. medication used to treat type-2 diabetes mellitus) on day 21 (*p* > 0.05). It may be suggested that the phytochemicals from both extracts of *A. bracteosum* could stimulate insulin release from the remaining pancreatic beta-cells and the enhanced potential regeneration of these cells in STZ-induced diabetic mice. These phytochemicals in other plants have previously been observed to possess significant antioxidant and hypoglycemic activities [33,34].

The use of phytochemicals provides a promising approach for many diseases [35,36,37,38,39] due to their anti-inflammatory, antioxidative and anticholinesterase activities. Regarding the antioxidant, α-glucosidase inhibition, and anti-hyperglycemic activities, the ethanol extracts of the plant exhibited more activity than the aqueous extracts. Thus, it is suggested that the isolated and identified phytochemical compound might be one of the main bioactive constituents from ethanol extract; and the isolated compound was identified as a flavonoid compound named isoscutellarein 8-O-β-D-glucopyranoside (IG). This is the first report on the occurrence of IG in *A. bracteosum*. This isolated compound was a strong inhibitor of α-glucosidase, being more than ten times higher than those of the acarbose drug and both extracts (Table 3). The anti-hyperglycemic effect of IG was effectively similar to the standard drug, glibenclamide, at the same dose of 10 mg/kg (*p* > 0.05), while groups receiving both extracts at dose of 50 mg/kg were equivalent to the drug group (Table 4). The IG compound had stronger α-glucosidase inhibitory and anti-hyperglycemic effects than those of both extracts, but the ethanol extract showed higher DPPH and ABTS free radical scavenging activities than that of IG (Table 3). To the best of our knowledge, the polarity and solubility of the isolated compound in the polar solvent, which was used in the DPPH and ABTS assays, may react with free radicals, and thus could affect the results. However, the IG compound showed a significant effect in DPPH and ABTS free radical scavenging. The present study provides the first measurement of the α-glucosidase inhibitory and anti-hyperglycemic properties of isoscutellarein 8-O-β-D-glucopyranoside, which possessed a more significant action than that of both extracts. These finding suggested that *A. bracteosum* could reduce the postprandial glucose by inhibiting the α-glucosidase activity. The extracts and the bioactive compound isolated from this plant demonstrated valuable pharmacological evidence, and could be used in the development of new antidiabetic drugs.

## 4. Conclusions

We demonstrated the antioxidant, anti-hyperglycemic, and α-glucosidase inhibitory activities of the extracts and bioactive compound from *A. bracteosum*. The results obtained in the present study strongly indicate that *A. bracteosum* is a potential source of natural anti-hyperglycemic agents, which could be further developed into an anti-diabetic drug.

## Figures and Tables

**Table 1:** Total phenolic and flavonoid contents of *A. bracteosum* extracts.

Samples	Total Phenolic Content (mg GAE/g extract)	Total Flavonoid Content (mg RE/g extract)
Ethanol extract	241.47 ± 4.47 ^a^	101.99 ± 4.48 ^a^
Aqueous extract	225.91 ± 2.54 ^b^	26.13 ± 0.76 ^b^

Results were expressed as mean ± standard deviation (SD) of three experiments. In each column, different letters mean significant different (*p* < 0.01).

**Table 2:** Antioxidant activity of *A. bracteosum* extracts and IG determined by DPPH and ABTS. assays.

Samples	DPPH Assay IC_50_ (μg/mL)	ABTS Assay IC_50_ (μg/mL)
Ethanol extract	6.61 ± 0.17 ^b^	10.33 ± 0.10 ^b^
Aqueous extract	10.12 ± 0.13 ^c^	11.17 ± 0.05 ^c^
IG compound	11.93 ± 1.23 ^d^	11.41 ± 0.63 ^c^
Standard (ascorbic acid)	2.89 ± 0.09 ^a^	5.25 ± 0.62 ^a^

Results were expressed as mean ± SD of three experiments. In each column, different letters mean significant different (*p* < 0.01).

**Table 3:** α-glucosidase inhibitory activity of the both extracts and IG compound.

Samples	α-Glucosidase Inhibition IC_50_ (μg/mL)
Ethanol extract	26.55 ± 1.57 ^b^
Aqueous extract	42.12 ± 3.02 ^c^
IG compound	1.40 ± 0.19 ^a^
Acarbose	87.94 ± 4.08 ^d^

Results were expressed as mean ± SD of three experiments. In each column, different letters mean significant different (*p* < 0.01).

**Table 4:** Effects of ethanol and aqueous extract of *A. bracteosum* on blood glucose.

Treatment	Dose	Blood Glucose (mmol/L)	% Inhibition (Compared with Group II)
Normal group (blank control)	Not observed	6.42 ± 0.47 ^cd^	Not observed
Group I (Glucose)	2 g/kg	12.05 ± 0.62 ^a^	Not observed
Group II (Glibenclamide)	10 mg/kg	5.91 ± 0.38 ^d^	56.68
Group III (aqueous extract)	40 mg/kg	7.66 ± 0.29 ^b^	36.43
Group IV (aqueous extract)	50 mg/kg	5.88 ± 1.26 ^d^	51.20
Group V (ethanol extract)	40 mg/kg	6.92 ± 0.38 ^c^	42,57
Group VI (ethanol extract)	50 mg/kg	5.48 ± 0.40 ^d^	54.52
Group VII (IG)	10 mg/kg	6.35 ± 0.11^cd^	47.30

Group I: normal control mice receiving water; Group II: glucose-induced diabetic mice treated with water; and the Groups III–VII: glucose-induced diabetic mice were treated with the extract accordingly. Data were mean ± SD of values from six mice. In each column, different letters mean significant differences (*p* < 0.05).

## Supplementary Materials

The following are available online at https://www.mdpi.com/2218-273X/10/2/201/s1: Figure S1: ^1^H NMR spectrum of compound IG in DMSO-d_6_ at 500 MHz.; Figure S2: ^13^C NMR spectrum of compound IG in DMSO-*d*_6_ at 125 MHz.; Figure S3: HMBC spectrum of compound IG in DMSO-*d*_6_.; Figure S4: HSQC spectrum of compound IG in DMSO-*d*_6_.; Figure S5. HR-ESI-MS spectrum of compound IG.
