# Supplementary material for: Potential Antidiabetic Activity of Extracts and Isolated Compound from Adenosma bracteosum (Bonati)
Source: Biomolecules. 2020 Jan 29;10(2):201. doi: 10.3390/biom10020201 (PMC7072461; doi:10.3390/biom10020201)

Supplementary Data

ISOLATION AND EVALUATION ANTIDIABETIC ACTIVITY OF *Adenosma bracteosum* (Bonati)

Legend of Figure

Figure S1. ^{1}H NMR spectrum of compound IG in DMSO-d_6 at 500 MHz
Figure S2. ^{13}C NMR spectrum of compound IG in DMSO-d_6 at 125 MHz
Figure S3. HMBC spectrum of compound IG in DMSO-d_6
Figure S4. HSQC spectrum of compound IG in DMSO-d_6
Figure S5. HR-ESI-MS spectrum of compound IG

Figure S1. ^{1}H NMR spectrum of compound IG in DMSO-d_6 at 500 MHz

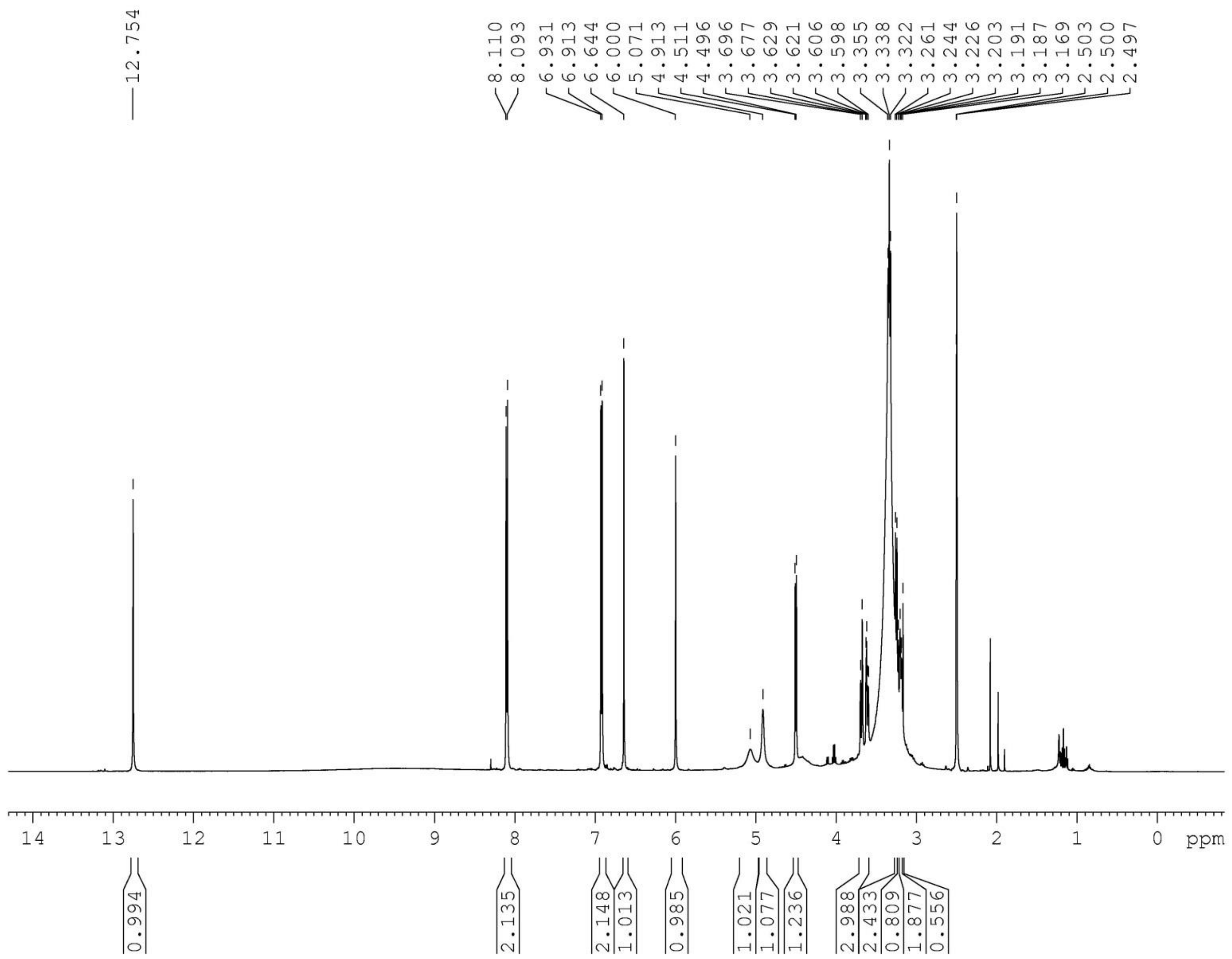

Figure S2. ^{13}C NMR spectrum of compound IG in DMSO-d_6 at 125 MHz

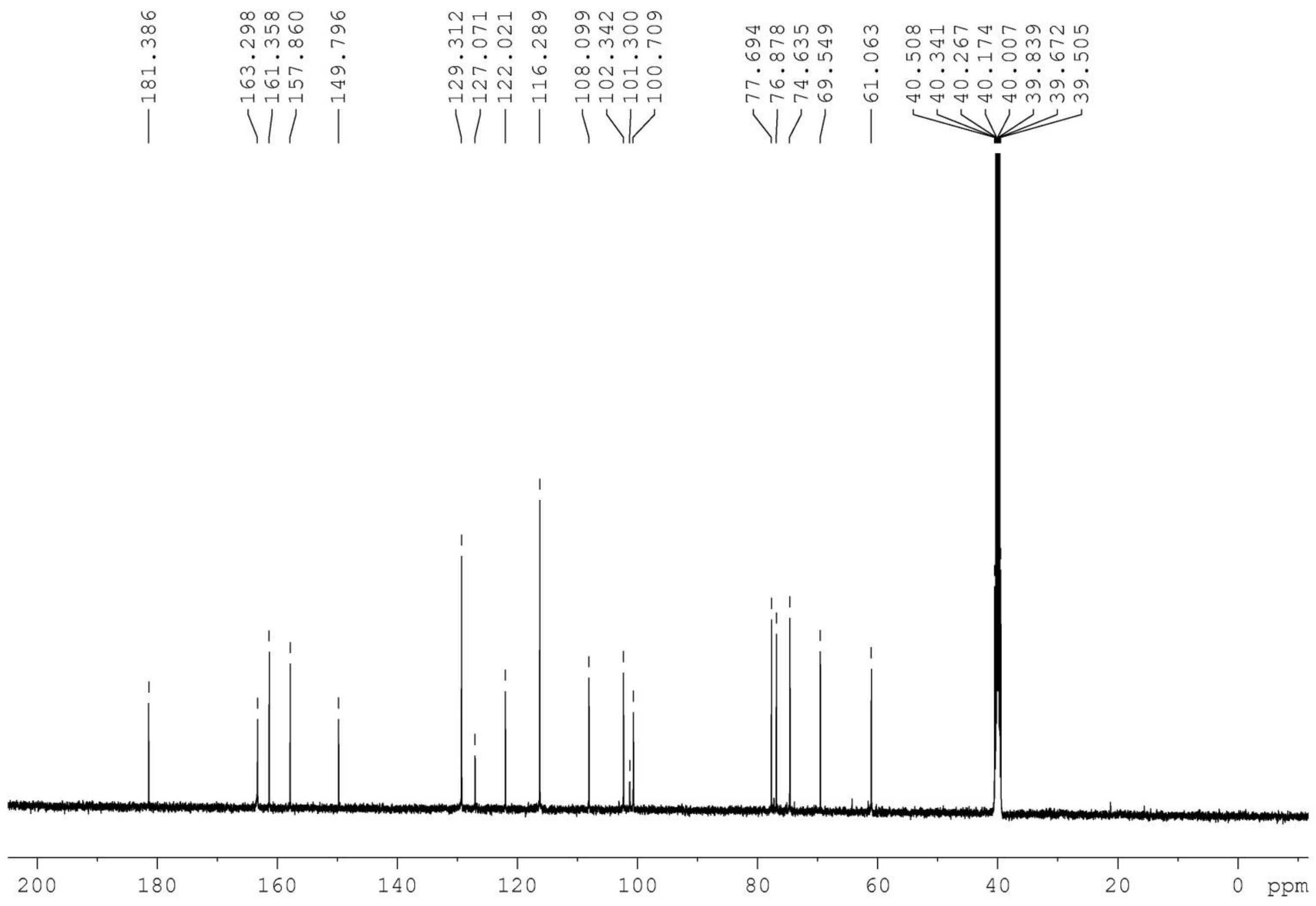

Figure S3. HMBC spectrum of compound IG in DMSO-d_6

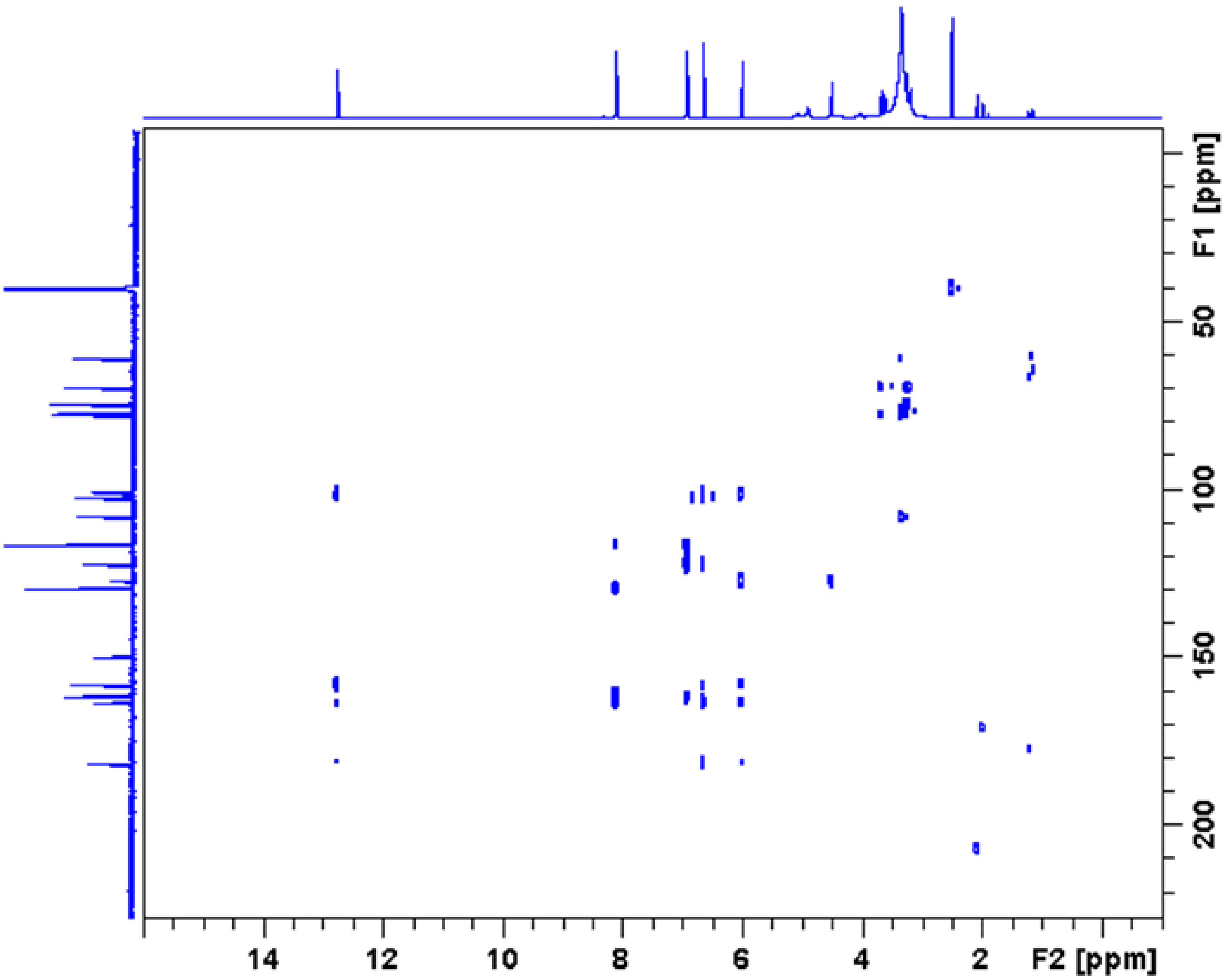

Figure S4. HSQC spectrum of compound IG in DMSO-d_6

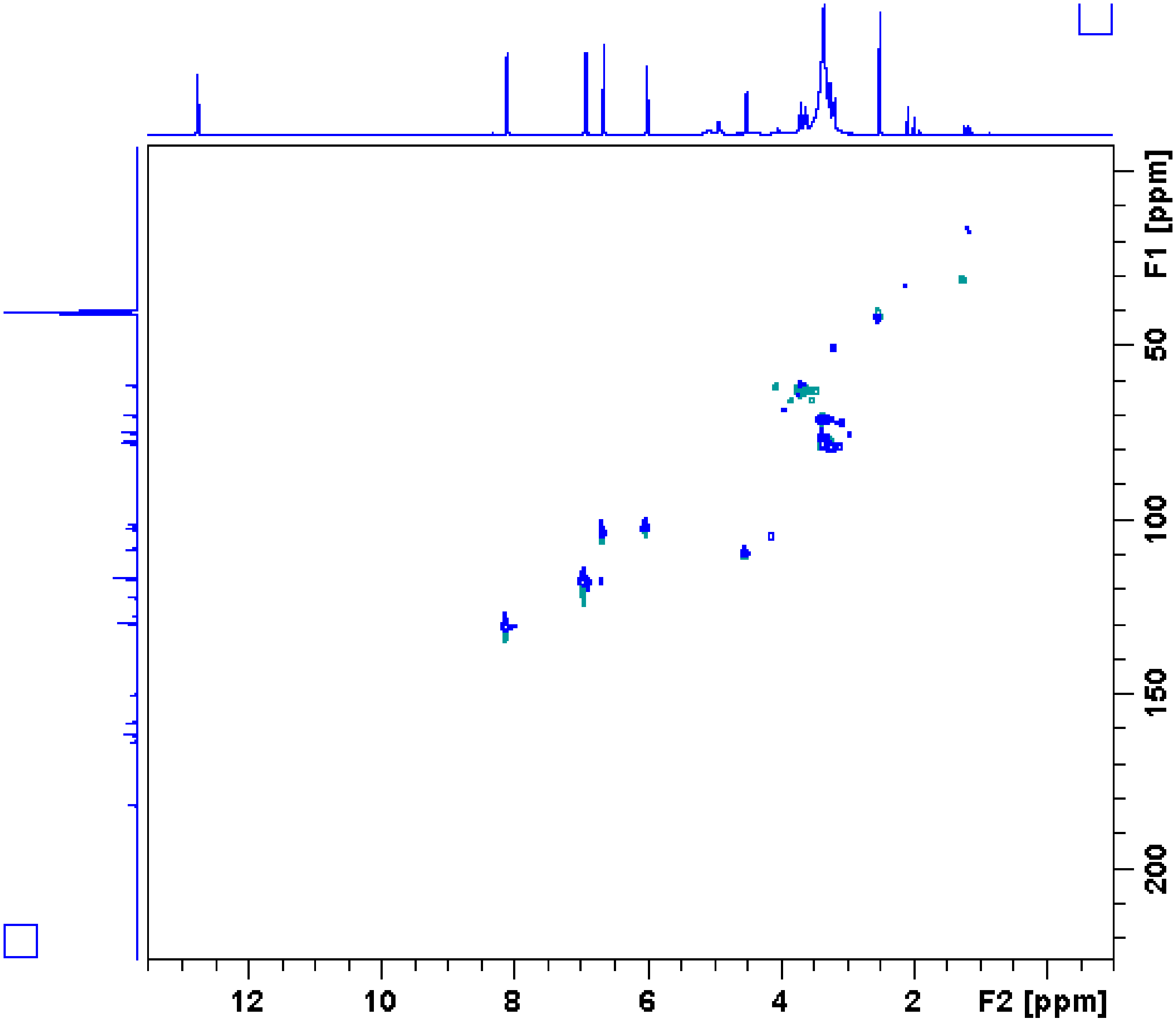

Figure S5. HR-ESI-MS spectrum of compound IG

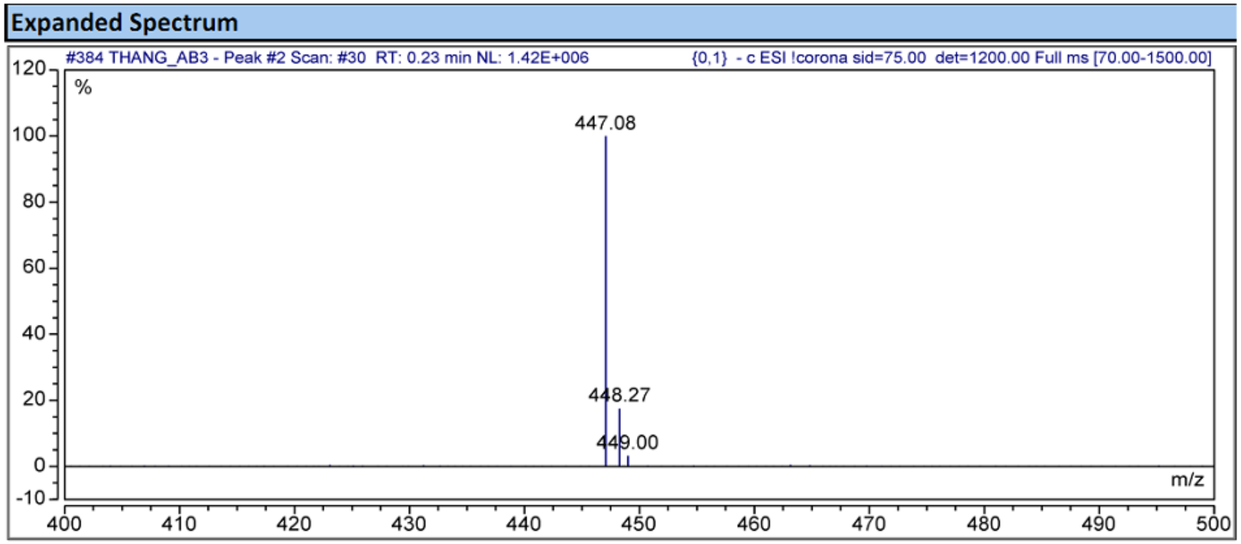

Supplement: Supplementary file 1 [file biomolecules-10-00201-s001.pdf]
